# An Argument for the Foundations of Population Mental Health

**DOI:** 10.3389/fpsyt.2018.00600

**Published:** 2018-11-19

**Authors:** Laura Sampson, Sandro Galea

**Affiliations:** Department of Epidemiology, School of Public Health, Boston University, Boston, MA, United States

**Keywords:** population health, public health, mental health, research, and macrosocial determinants of health

## How we see the world

The global burden of disease has shifted from the infectious diseases of the nineteenth and early twentieth century toward a growing burden of chronic illness (1). Our understanding of disease causation has commensurately shifted over time. Our original study of infectious diseases necessitated isolating specific causes; this was embedded most clearly perhaps in Koch's postulates that served as some of the earliest grounding for causal thinking in population health science (2). However, as chronic disease has become much more pervasive, it is clear that a focus on isolating individual causes is insufficient to explain more complex disease etiologies. By way of illustration, it is clearly inadequate to describe the dramatic rise in obesity worldwide as a function solely of our genes; any comprehensive causal approach must take into consideration changing dietary patterns and food availability.

That we fall short in turning our attention to a more complex view of disease causation was argued perhaps most elegantly by McMichael in a seminal paper at the turn of the century (3). McMichael suggests that preoccupation with individual genetic or behavioral factors falls short of the larger socio-ecologic context. If we are to extend beyond proximate risk factors, McMichael argues, we must broaden our causal models and learn to apply a social-ecologic systems perspective. While McMichael's work was aimed at chronic physical diseases, his admonition has relevance and applicability to our thinking about population mental health today.

## Our approach to mental health, and its consequences

Mental health research remains primarily centered on isolating single causes, often by targeting subgroups that can help us identify causes when taking into account potential confounders. For example, in cohort studies, we take samples of exposed and unexposed people and follow them over time to see who develops disease (4, 5), while in case-control studies, we take samples of diseased and non-diseased people and compare exposure status (4, 6). We do so even in population-based cross-sectional studies in which we attempt to randomly sample the population in order to assess prevalence (7, 8). The body of mental health research typically isolates specific subgroups without paying much attention to whole populations from which they originate.

This preoccupation with identifying individual risk factors is further compounded by modern advances in biology. In recent years, funding for academic research has been disproportionately awarded to studying genetic risk factors compared to the proportion of common diseases actually attributed to genetic differences (9). For example, according to the National Institutes of Health's current active projects total as of December 2017 (10), projects with the word “genetic(s)” in the project title, terms or abstract account for 39% of the total currently funded projects ($15.8 billion out of $40.4 billion). For the funds coming out of the National Institute of Mental Health specifically, the proportion is the same ($711 million out of $1.8 billion).

To highlight a specific mental disorder by way of illustration, the 2014-2015 research portfolio report for Autism Spectrum Disorder (ASD) (11) shows the that the breakdown of funding by sub- category for risk factors of ASD highly favors gene-related studies, with only 16% of funding in 2014 and 13% of funding in 2015 allocated toward studying environmental risk factors, and the remaining toward studies of genetic risk factors, epigenetics, or gene-environment interactions.

In part as a result of funding being made more readily available for genetic studies in recent years, and in part as a reaction to recent interest in the field of “personalized” or “precision medicine” (12, 13), the landscape of health-related publications has changed over the last decade. As an illustration of this shift, we searched the PubMed database (14) for articles published across the past 15 years (indexed as of December 13, 2017). We recorded the number of total publications by year; the number with the phrase “population health” in the title or abstract; and the number with the phrases “personalized [personalized] medicine,” “individualized [individualized] Medicine,” “individual medicine,” or “precision medicine” in the title or abstract. We then divided the number in the former category by the total for each year, and the number in the latter category by the total for each year, in order to estimate proportions of total publications centering on each of these areas over time.

**Figure 1:**
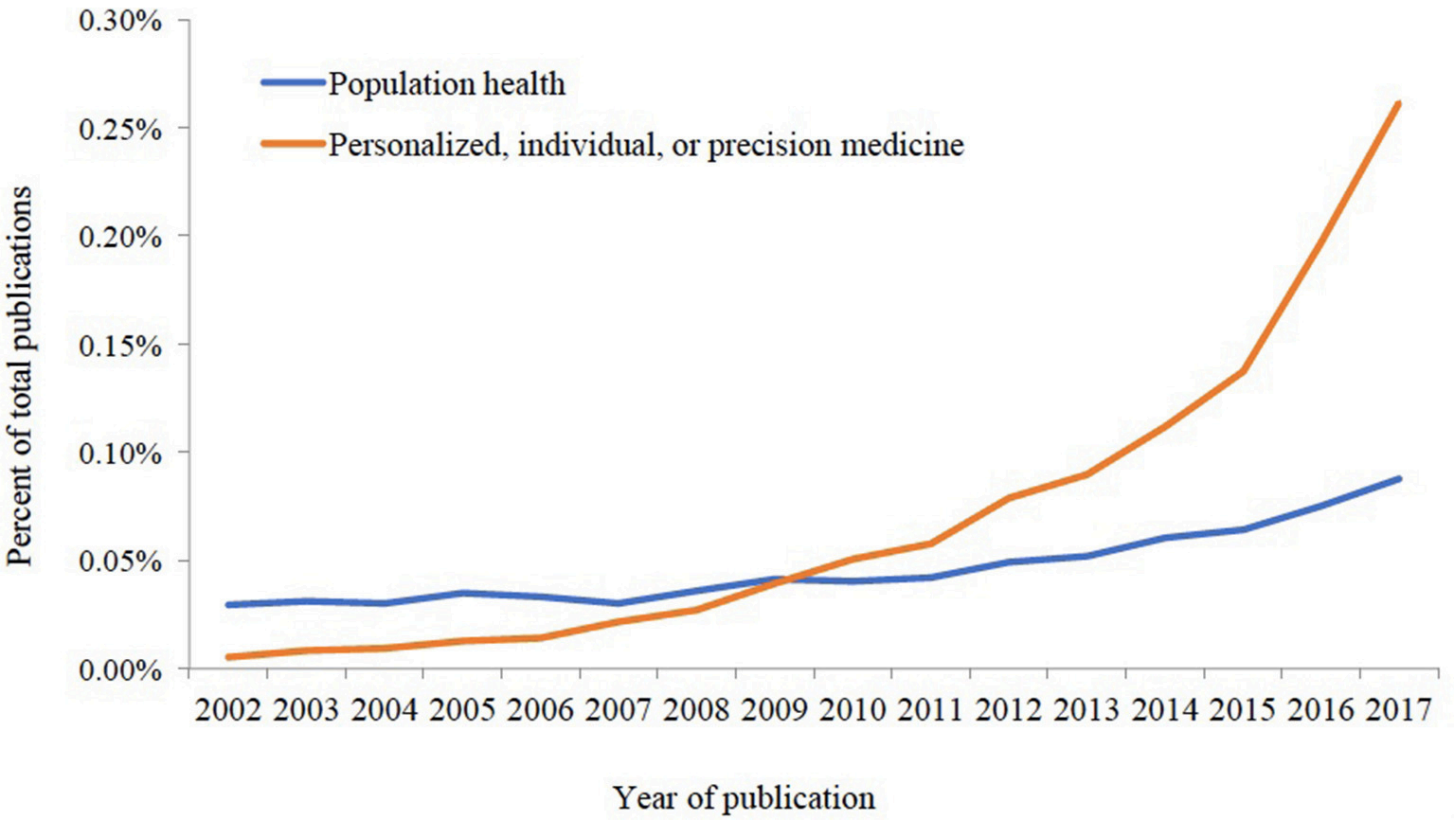
Proportion of publications indexed in PubMed, 2002–2017 with relevant phrases included in the title or abstract.

As one can see in Figure 1, the proportion of papers mentioning population health has stayed relatively stable over time, increasing by a small amount in the last few years. Papers mentioning personalized, individual or precision medicine, by comparison, have increased tremendously as a proportion of the overall number of publications in PubMed. The proportion of publications explicitly dealing with precision medicine in 2017 (0.26%) is about 50 times that in 2002 (0.005%), and about three times the proportion of publications mentioning “population health” in 2017 (0.088%).

There is little doubt about the importance of identifying and better understanding the genetic determinants of mental health; we have tremendous hope for the potential of discovery science in the long-term. The challenge lies in an undue attention toward isolating causes and eliding population-level thinking about what produces health in populations. At core, ignoring population-level drivers results in non-reproducibility of results that have at best identified causes in small, particular samples. For example, a recent meta-analysis (15) found no significant associations between depression and the serotonin transporter gene (5-HTTLPR) across 14 studies with a total of 14,250 participants, nor did it find an interaction between the gene and stressful life events.

Similarly, the Major Depressive Disorder (MDD) group of the Psychiatric Genome-wide Association Study Consortium reported that, “although this is the largest genome-wide analysis of MDD yet conducted…we were unable to identify robust and replicable findings” (16). There are many potential reasons for such issues of reproducibility in genetic studies of mental health outcomes. One explanation could stem from inherent uncertainty of psychiatric diagnoses, including the lack of biomarkers for disorders such as depression and an often unclear dichotomy between case status and non-case status, making validity of outcomes across different studies difficult to measure and standardize. However, we argue that unmeasured aspects of the larger environment are likely the greatest contributor to challenges in reproducibility of studies focused on individual-level effects (17). This problem calls for a reexamination on how we approach the drivers of population mental health.

## A different approach

The issue of reproducibility brings us to a potential solution: returning to the focus on whole populations that was crucial to the founding of the public health field in the first place.

Population health science is a burgeoning field that may have utility for mental health researchers and practitioners. Population health science is concerned with the conditions that shape distributions of health within and across populations, and of the mechanisms through which these conditions manifest as the health of individuals (18). Population health science encourages a focus on exposures that are pervasive, ubiquitous, and hence difficult to study. Such exposures may be referred to as macrosocial factors; examples include social or cultural norms, urbanization, discrimination, political structures, air pollution, poverty, climate change, or migration patterns; these are all forces that surround us and are primarily “invisible” even as they have profound implications for the determination of population health.

We can perhaps think of the foundational drivers of population mental health through an analogy of the water in a goldfish bowl. Similar to how the water is invisible to the goldfish, we are often unaware of, or ignore, these foundational determinants of health in our environment. Ignoring such core drivers of health that shape systems and norms is akin to ignoring the overall water quality of a goldfish bowl, instead only studying differences in health among individual goldfish within the bowl. When we do this, we are missing key opportunities. In fact, small changes in macrosocial factors often result in more substantial changes in the health of populations than larger changes in rarer causes (18).

We will illustrate this point through two examples. First, centering in on one of these foundational factors, Jackson and Knight suggest a causal pathway linking the stress of discrimination to an increase in negative health behaviors such as smoking and alcohol abuse as coping mechanisms, that then interfere with stress response and result in mental disorders (19). This pathway suggests that if we could intervene on the more upstream, structural forms of discrimination instead of on the behaviors that often follow it, we may be more successful in reducing disparities in mental health, among other outcomes.

Second, to use a numerical example, suppose we have a population that experiences 450 cases of suicide out of 51,000 over a 5-year period. Suppose further that this population has pervasive income inequality, which is associated with a modest 1.25 times increased risk of suicide and risk difference of 0.0018, but which affects everyone in our population. Let us say that exposure to an extremely traumatic event within this population is also associated with suicide, with a larger risk ratio of 6.25 and risk difference of 0.042, but the traumatic event only has a prevalence of 1.96% in the population (therefore, there are 50 cases in the exposed group and 950 cases in the unexposed group). One might think that because the effect of the traumatic event is of a much higher magnitude than that of income inequality, that we might avoid more cases of suicide by eliminating the traumatic event than by eliminating income inequality.

Conversely, if we keep the prevalence of the traumatic event at 1.96%, while reducing suicides by a factor of 1.25 in the entire population (among both the groups exposed to the traumatic event and those unexposed to the traumatic event), we end up with 360 total cases of suicides. Thus, we “save” 90 lives (450–360 = 90) by removing a ubiquitous cause. By comparison, according to the risk difference, if we were to remove traumatic events from the population, we would only “save” 4.2 lives per 100 persons exposed to the traumatic event, or a maximum of 42 lives out of the 1,000 exposed in our population (18). Because the traumatic event was a rarer exposure, it was associated with fewer deaths avoided than the weaker, but ubiquitous, cause of income inequality.

Might this approach work outside the hypothetical? We argue that past public health successes such as decreased motor vehicle mortality due to seatbelt laws, or changing smoking norms—neither of which seemed possible at a time—illustrate that by focusing on foundational factors we can indeed shift the health of populations effectively.

Importantly, we argue that reckoning with these foundational factors is non-discretionary. We estimated the number of deaths attributable to social factors based on a systematic review of the literature, and found that about 245,000 deaths in the United States in 2000 were attributable to low education, for example, while 176,000 were attributable to racial segregation and 162,000 to low social support (20). It is important to note that these types of foundational determinants of health do not only cause disease in those who are considered “high risk.” Rather, these foundational conditions also affect those who are at “low risk,” effectively driving health in whole populations, making them ineluctable drivers of health (21).

## Intervening to change foundational determinants of health

One of our conceptual challenges in tackling the foundational drivers of health is that interventions are harder to implement. In many respects, however, that is as much a failure of our imagination as it is of practical concern. Examples of successful population-level interventions are available and hold promise. The Moving to Opportunity Study in the 1990's, for example, randomized low-income families living in public housing projects to receive housing vouchers for higher-income areas, in order to measure the effects of living in a low-income neighborhood on various outcomes (22). Families who moved to higher-income neighborhoods experienced lower rates of distress among parents and lower rates of depressive/anxious symptoms and dependency problems among male children, compared to families who stayed in public housing (23), suggesting measurable effects of neighborhood-level determinants of mental health which may be modifiable.

One could imagine that if we as a field encouraged similar types of large-scale interventions that address pervasive issues such as income inequality, at least as much as we prioritize individualized approaches such as focusing on genetic risk factors, we might have a better chance of affecting changes in incidence of mental health related events. An example of a potentially fruitful population-wide intervention for mental health-related problems is reduction in firearm access. Studies in other countries have shown that reducing access to firearms was associated with lower suicide rates among adolescents (24), and one state-level analysis completed by our group showed that within the U.S., states with universal background checks and states with ammunition background checks each had significantly lower firearm-related mortality compared to states without these laws (25). If implemented nationally within the U.S., these policy-level interventions would affect all people, not only high-risk individuals such as those with depression or demonstrated suicidality, and would likely have wide-reaching effects in a country with such high rates of firearm mortality. A population health approach like this, shifting the overall curve of exposure, as opposed to targeting only high-risk individuals—an idea that was introduced decades ago by Geoffrey Rose—can be transformative for population mental health (26).

## Conclusion

We argue here for a population health strategy applied, in particular, to mental health concerns. This strategy is not a new one. In some ways, this approach takes us back to the industrial revolution, during which public health practitioners fought for large-scale, population-wide solutions to overcrowding, work conditions, and poor hygiene in order to improve individual health. We suggest, however, that it is time to bring this large-scale focus on populations to the field of mental health, in the midst of the precision medicine era. Importantly, we suggest that confronting social, political, and economic factors (27) is essential to any effort that aims to improve population health, making this conceptual shift indeed overdue.

## Author contributions

LS wrote the manuscript with input and ideas from SG. SG critically edited all versions of the manuscript. Both authors contributed to and stand behind all aspects of the work.

### Conflict of interest statement

The authors declare that the research was conducted in the absence of any commercial or financial relationships that could be construed as a potential conflict of interest.
